# Escher: A Web Application for Building, Sharing, and Embedding Data-Rich Visualizations of Biological Pathways

**DOI:** 10.1371/journal.pcbi.1004321

**Published:** 2015-08-27

**Authors:** Zachary A. King, Andreas Dräger, Ali Ebrahim, Nikolaus Sonnenschein, Nathan E. Lewis, Bernhard O. Palsson

**Affiliations:** 1 Department of Bioengineering, University of California, San Diego, La Jolla, California, United States of America; 2 Center for Bioinformatics Tuebingen (ZBIT), University of Tuebingen, Tübingen, Germany; 3 Novo Nordisk Foundation Center for Biosustainability, Technical University of Denmark, Lyngby, Denmark; 4 Department of Pediatrics, University of California, San Diego, La Jolla, California, United States of America; University of Canterbury, NEW ZEALAND

## Abstract

Escher is a web application for visualizing data on biological pathways. Three key features make Escher a uniquely effective tool for pathway visualization. First, users can rapidly design new pathway maps. Escher provides pathway suggestions based on user data and genome-scale models, so users can draw pathways in a semi-automated way. Second, users can visualize data related to genes or proteins on the associated reactions and pathways, using rules that define which enzymes catalyze each reaction. Thus, users can identify trends in common genomic data types (e.g. RNA-Seq, proteomics, ChIP)—in conjunction with metabolite- and reaction-oriented data types (e.g. metabolomics, fluxomics). Third, Escher harnesses the strengths of web technologies (SVG, D3, developer tools) so that visualizations can be rapidly adapted, extended, shared, and embedded. This paper provides examples of each of these features and explains how the development approach used for Escher can be used to guide the development of future visualization tools.

## Author Summary

We are now in the age of big data. More than ever before, biological discoveries require powerful and flexible tools for managing large datasets, including both visual and statistical tools. Pathway-based visualization is particularly powerful since it enables one to analyze complex datasets within the context of actual biological processes and to elucidate how each change in a cell effects related processes. To facilitate such approaches, we present Escher, a web application that can be used to rapidly build pathway maps. On Escher maps, diverse datasets related to genes, reactions, and metabolites can be quickly contextualized within metabolism and, increasingly, beyond metabolism. Escher is available now for free use (under the MIT license) at https://escher.github.io.

This is a *PLOS Computational Biology* Software Article.

## Introduction

The behavior of an organism emerges from the complex interactions between genes, proteins, reactions, and metabolites. With next-generation sequencing and various “omics” technologies, it is now possible to rapidly and comprehensively measure these components and interactions. These technologies have transformed the scientific process over the past decade. Data acquisition is substantially easier, but data analysis is increasingly becoming the primary bottleneck to discovery. To address the analysis bottleneck, there has been a demand for data visualization tools to complement statistical and modeling methods.

Biological visualizations often fall into categories characterized by biological scale, and the style of a visualization reflects the type of information at that scale. Three-dimensional objects are often used for representing protein structures [1, 2], one-dimensional tracks for genome sequences [3, 4], force-directed graphs for interaction networks [5], trees for phylogenetic relationships [6, 7]. And, finally, two-dimensional *pathway maps* have long been a popular visual representation of metabolic pathways and other biological pathways. For each type of visualization, data can be associated with the biological components in the visualization. Visualizing data in this way contextualizes and enriches the dataset for scientists. Data-rich visualizations have been extremely valuable for viewing, interpreting, and communicating data.

A tool for visualizing pathway maps must satisfy a set of core features. The tool must (1) visually represent reactions and pathways clearly and in a way that is biochemically correct, (2) allow users to navigate and search through the visualization, (3) allow users to design and customize pathway maps, (4) allow users to represent diverse data types within the map using visual cues like size and color, (5) provide import and export features so that maps can be stored, shared, and exported to other tools, and (6) provide an application program interface (API) so the tool can be used within data analysis pipelines.

The existing tools that satisfy these core features are all desktop applications. Briefly, these tools include Omix [8], Cytoscape [5], CellDesigner [9], Vanted [10] with the SBGN-ED add-on [11], VisAnt [12] and PathVisio [13]. Desktop applications have many advantages over web applications, including speed, stability, and integration with the operating system, and these merits have made desktop applications more popular.

The advantages of web applications include rapid deployment (no need to download an application or browser plug-in), greater cross-platform compatibility (e.g. mobile devices), flexible sharing, collaborating, and embedding features, as well as easy application development. Recently, a critical mass of performance enhancements and new libraries has made web tools comparable to desktop tools for many applications.

A number of web-based tools exist for visualizing pathway maps: ArrayXPath [14], Pathway Projector [15], iPath2.0 [16], WikiPathways [17], Biographer [18], and the BioCyc pathway viewer [19]. However, none of these satisfy all the core features for a pathway map visualization tool.

One of the key differentiating features of a web application is that modern web browsers come with a built-in software development platform (often called the Developer Tools). This development platform includes a JavaScript shell for directly interacting with the web page runtime and a tool for inspecting and modifying every element in the web page document object model (DOM). Thus, *any user can locally modify any element on the page at any time*. If a web application is built on the DOM, then users can rapidly prototype new features and build extensions to the application while it is running. (A comparable feature is the extensibility of the EMACS editor, which can be extended while the editor is running [20]. On the strength of this feature, EMACS has remained popular for 30 years.) To utilize this powerful feature, one must use a visualization library that is based on the DOM, the most popular of which is Data-Driven Documents (D3) [21].

Escher is a web application for visualizing pathway maps, and it is designed to be a fully featured pathway visualization tool that also harnesses all the advantages of the web. Escher has three key features that distinguish it from all existing pathway visualization tools, including the popular desktop applications. First, Escher makes building pathway maps fast and easy, using the information in datasets and genome-scale models to suggest pathways to the user—with this, pathway map design can be semi-automated. Second, Escher connects genes and enzymes to the reactions they catalyze, so that genomic data can be visualized in the context of the reaction network. We show how Escher can be used to visualize reaction data (metabolic fluxes), metabolite data (metabolomics), and genomic data (transcriptomic data), bridging the gap between these data types. Third, Escher uses the advantages of web technologies so that pathway maps can be adapted, extended, shared, and embedded. We illustrate the export and development features of Escher, including native support for scalable vector graphics (SVG) export, a downloadable tool for converting Escher maps to common standards for representing layouts, and application program interfaces (APIs) for developing new applications that extend the functionality of Escher.

## Results

### Building pathway maps

To build a pathway map, one first needs a source for the names, stoichiometries, and associated genes for each biochemical reaction in an organism. This information is provided by a *c*onstraint-*b*ased *r*econstruction and *a*nalysis (COBRA) model, a collection of all the reactions, metabolites, and genes known to exist in an organism (also called a genome-scale model (GEM) or constraint-based model (CBM)) [22]. While COBRA models have generally focused on metabolism, the COBRA modeling approach can be applied to any biochemical reaction network [22], so Escher could be used to visualize pathways like gene expression and membrane translocation, which are now being incorporated into COBRA models [23–25].

**Fig 1:**
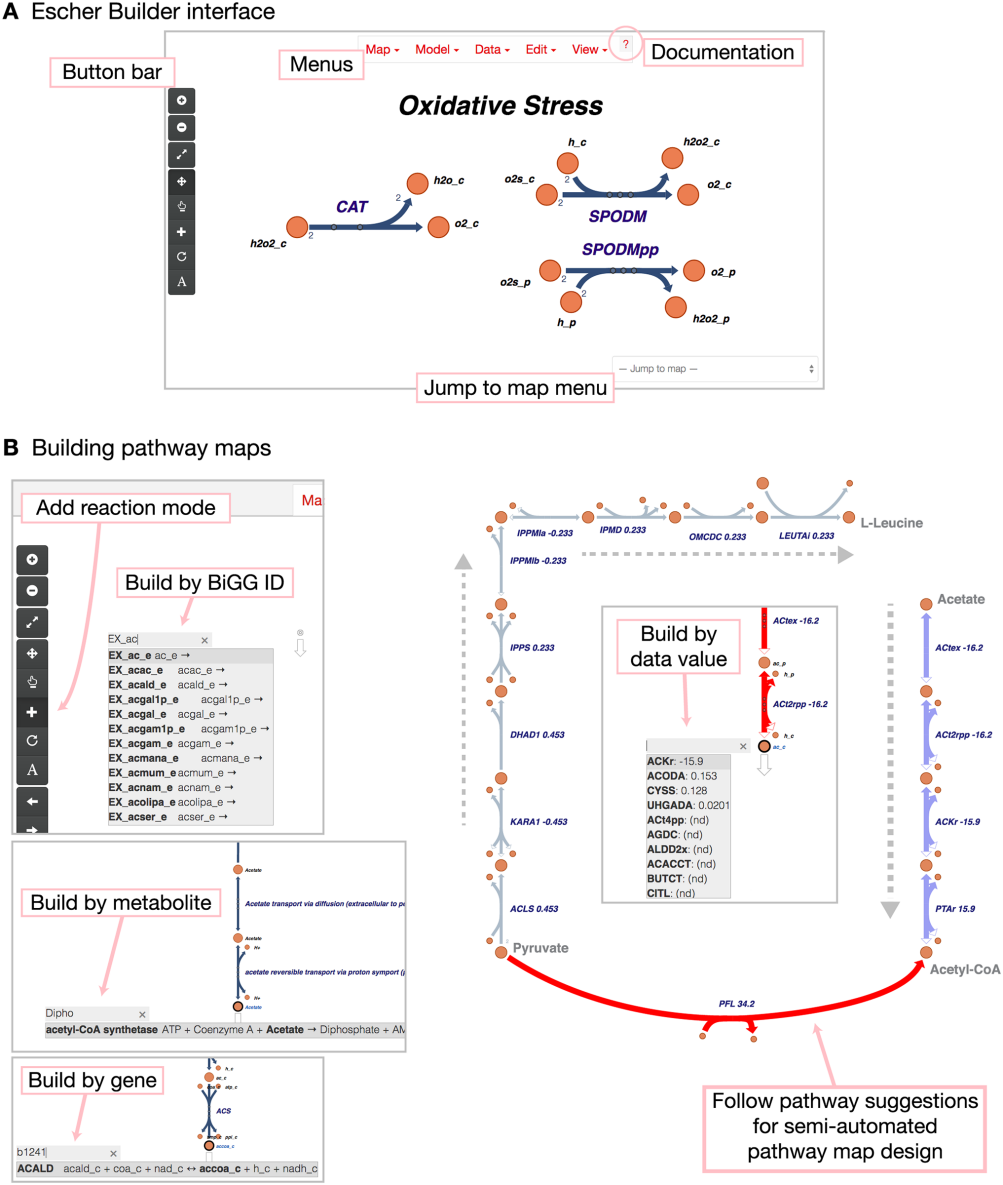
The Escher interface. A) The application includes a set of menus with a link to the documentation, a button bar for accessing common features, and a menu for jumping to maps that were built with the same model. B) To build pathway maps, enter the *Add reaction* mode using the **Edit** menu or the button bar. Click on the canvas or an existing metabolite to see a search menu. Reactions can be searched by reaction ID, by metabolite, and by gene. When a gene dataset or reaction dataset is loaded, suggestions appear for the reactions with the largest values in the dataset.

The Escher interface is centered around a canvas for the pathway map (Fig 1A). In the Escher Builder, a number of editing modes are available in the **Edit** menu; these include tools for navigating the map (Pan mode), selecting and modifying elements (Select mode), adding reactions (Add reaction mode), rotating the current selection (Rotate mode), and adding and editing text annotations (Text mode).

In *Add reaction mode*, a new pathway can be added to the canvas. Clicking on the canvas or an existing metabolite opens the new reaction search box. The search box can find reactions with a number of queries: reaction identifiers (IDs) and display names, metabolite IDs and display names, and gene IDs and names (Fig 1B). (IDs and names are based on those in the COBRA model.) If a reaction or gene dataset is loaded, then Escher provides suggestions of the next reaction to build, sorted by the data value for that reaction (Fig 1B).

With this set of suggestions, a user can quickly build an Escher map based on previous knowledge of the organism or using the suggestion of a dataset. Data-driven map layout is also extremely useful for understanding an organism at the genome-scale—guided by the data, it is possible to find all the elements of a network that are, for example, highly upregulated without any bias toward well known pathways. To add the top suggested reaction, a user can simply press the Enter key. Thus, if a pathway is linear or has high values in a given dataset, then pressing Enter repeatedly will draw a linear pathway that is based entirely on the information in the data and the COBRA model. This process can be repeated to build perpendicular branches from metabolites in the pathway.

The Escher interface includes a general menu, a menu bar for accessing common functions, a tool for switching between maps, and a canvas containing the interactive pathway map (Fig 1A). The **Map** and **Model** menus contain import and export functions for maps and COBRA models. The **Data** menu contains the data loading functions, and the **View** menu contains zoom options and access to the **Settings** page.

### Visualizing data

Three types of data can be visualized on an Escher map: reaction data, metabolite data, and gene data. And Escher supports visualizing a single dataset, or visualizing the comparison of two datasets using a number of comparison functions (log, log_2_, and difference). The **Settings** page includes a detailed set of options for coloring and sizing elements based on statistical features of a dataset (min, max, quartiles, mean). Here, examples are provided for each data type, and the files required for recreating the visualizations are in the supplementary data.

#### Reaction data

To demonstrate the visualization of reaction fluxes, an *in silico* simulation of anaerobic growth was performed in the *Escherichia coli* COBRA model *i*JO1366 using parsimonious flux balance analysis (pFBA) [26, 27]. The Escher map of *i*JO1366 central metabolism was loaded (iJO1366.Central Metabolism) and the dataset (S1 Data) was loaded using the **Data>Load reaction data** function. (Datasets can be JavaScript Object Notation (JSON) or comma separated values (CSV) files, as described in the documentation.) Two settings were changed for this visualization: The absolute value of reaction data was visualized so that negative fluxes appear as large values, and the secondary nodes were hidden to simplify the visualization.

**Fig 2:**
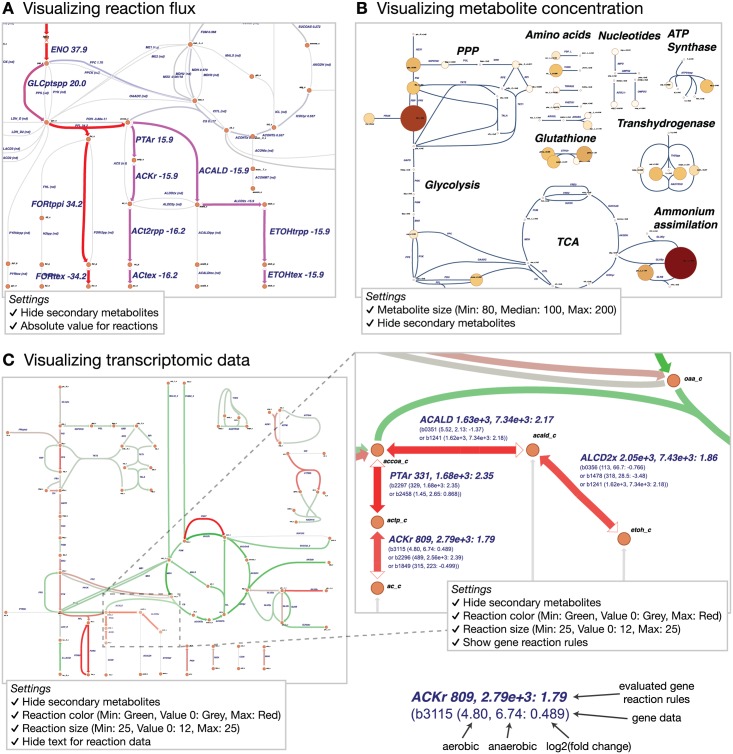
Data visualization. A) The results of an *in silico* flux simulation visualized on the reactions. B) Metabolomics data for *E. coli* aerobic growth visualized on the metabolites. C) RNA-Seq data showing the shift from aerobic to anaerobic conditions in *E. coli*. Green represents reactions downregulated in anaerobic growth and red represents gene upregulated in anaerobic growth, based on the log_2_ of the fold change.

The resulting figure shows reaction fluxes for fermentation pathways (Fig 2A). It was downloaded as a SVG image with the command **Map>Export as SVG**, and the text labels of the high flux reactions were made larger for the figure.

#### Metabolite data

Metabolite concentrations are shown from a dataset recently reported by our research group [28], which were organized in a CSV file with metabolite BiGG IDs as keys (S2 Data). The example figure shows aerobic metabolite concentrations on a modified map of *E. coli* central metabolism (S3 Data). To better identify metabolite concentration differences, the metabolite size was changed on the **Settings** page, and the secondary metabolites were hidden.

The resulting figure provides a high level view of the most abundant metabolites in the network during aerobic growth of *E. coli* (Fig 2B). It was downloaded as a SVG image with the command **Map>Export as SVG**, and the text annotations were made larger for the figure.

#### Gene data

To demonstrate the use of gene data on an Escher map, transcript abundances for aerobic and anaerobic growth of *E. coli* were calculated using RNA-Seq datasets from a recent publication [29]. The datasets were downloaded from the Gene Expression Omnibus (GEO) repository [30] (accession number GSE48324), and fragments per kilobase of exon per million fragments mapped (FPKMs) were calculated using the Cufflinks functions cuffquant and cuffnorm [31], with appropriate parameters for the library type of the published data. These data were then collected, with locus tags as gene identifiers, in a single CSV file (D4 Data).

To connect genomic data with the reactions on an Escher map, Escher must consider which gene products are responsible for catalyzing each biochemical reaction. This association can be defined using Boolean *gene reaction rules* (also called gene-protein-reaction associations (GPRs)) [32]. When either of two enzymes can catalyze a reaction—as with isozymes—then these genes are connected with an OR rule. Escher *adds* the values of two genes connected with an OR rule. When two enzymes are required together for catalysis—as in an enzyme complex—these are connected with an AND rule. Escher can take the *mean* or the *minimum* of the two values connected with an OR rule; this option is selected on the **Settings** page. For a comparison of two datasets, the gene reaction rules are evaluated for each dataset separately, then a comparison is made between the two resulting values (Fig 2C).

The resulting figure shows the shift from aerobic to anaerobic conditions, where green reactions are downregulated anaerobically and red reactions are upregulated anaerobically (Fig 2C). Escher shows the log_2_ of fold change between the conditions. However, Escher cannot yet display statistical significance for the datasets, so it should be paired with statistical tools (e.g. cuffdiff [31]).

## Design and Implementation

### JavaScript

**Fig 3:**
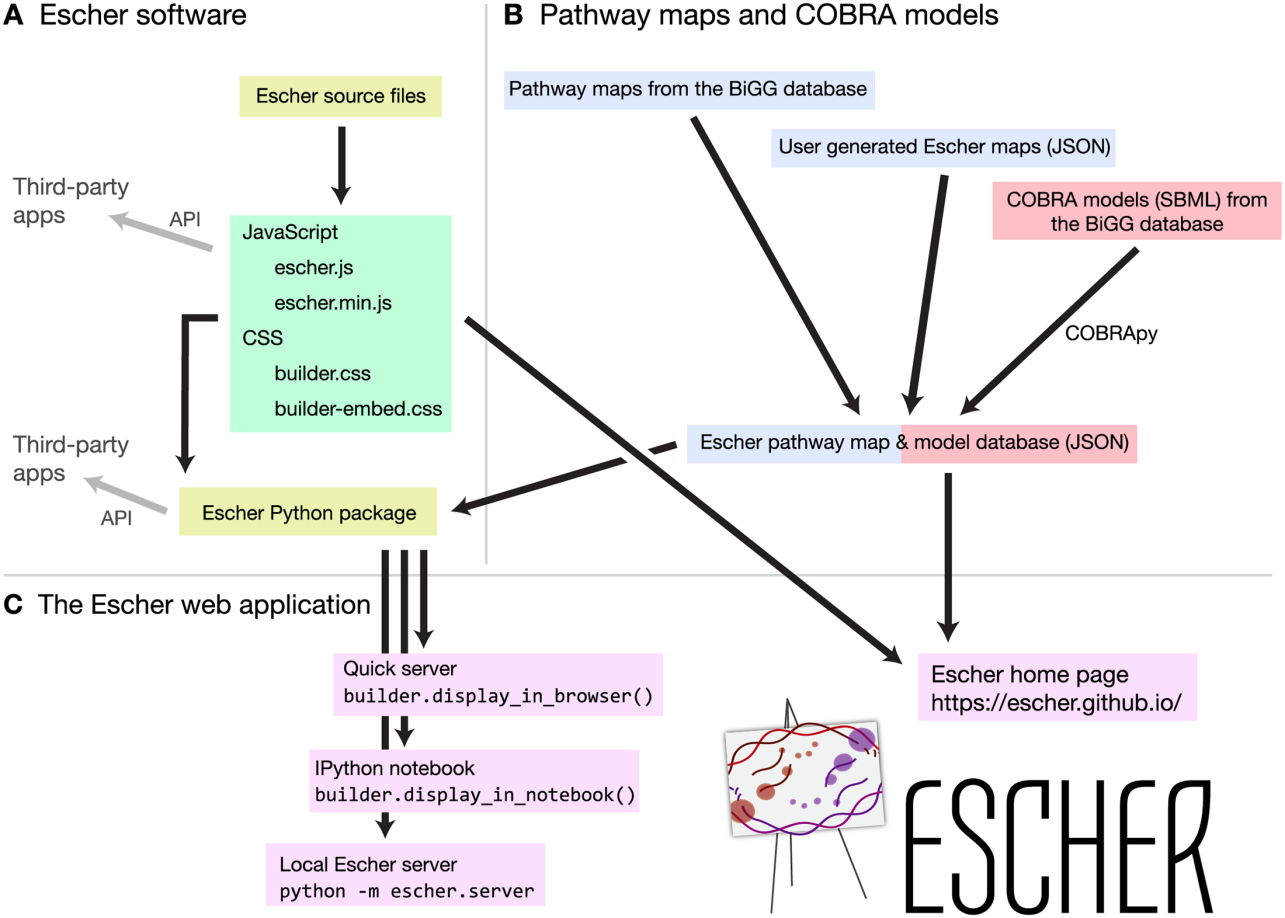
The organization of the Escher project. A) Escher source code can be compiled to a single JavaScript file (either minified or not minified) and two style sheets. The Python package is used to serve the Escher web application in various ways. APIs exist for both JavaScript and Python. B) Escher maps are generated from the BiGG database or built by users. COBRA models are generated using COBRApy. C) The Escher web application can be viewed on the Escher website, or, for local access, using various methods in the Python package.

Escher is a web application written primarily in JavaScript, using the libraries D3 [21], and, optionally, JQuery (http://jquery.com) and Bootstrap (http://getbootstrap.com). The Escher JavaScript code can be compiled into a single JavaScript file, and a JavaScript API is available for interacting with and extending an Escher visualization (Fig 3A). All layout, editing, import, and export features of Escher are included in the JavaScript library, and the default visual styles are defined in two cascading style sheets (CSS) files. The Escher website is built using the JavaScript API, and other web applications can be built on top of this library.

### Python

A Python package for Escher is also available (Fig 3A), and this package includes a number of extra features: access to Escher maps from Python terminals and IPython Notebooks, offline access to Escher, a local server with map and model caching, and a Python API for developing applications with these additional features. Accessing maps from Python and IPython Notebook allows Escher to be integrated directly with data analysis and modeling workflows. For example, within an IPython Notebook, the results of an *in silico* flux simulation can be applied to an Escher map, and the map will be embedded and shared with the notebook. Escher even supports NBViewer for sharing static IPython Notebooks as websites (http://nbviewer.ipython.org).

### Map and model database

Escher includes a database of pathway maps and genome-scale models. Pathway maps are currently available for a number of organisms, and new pathway maps will be continually added to the database from our group. The maps in the BiGG database are being converted to the new Escher format [33]. We also accept contributions from the community, and the method for submitting pathway maps is described in the documentation (S2 File).

### JSON schema

Both Escher maps and COBRA models are stored as JavaScript Object Notation (JSON) files. JSON is a useful, plain-text format for storing nested data structures. For Escher maps, a JSON Schema has been defined (S1 File, see the schema file escher/jsonschema/1-0-0), and the schema can be enforced using the JSON Schema validators available in a number of languages (http://json-schema.org). Thus, Escher maps conform to a well-defined specification that can be generated by other tools and scripts.

### Export

**Fig 4:**
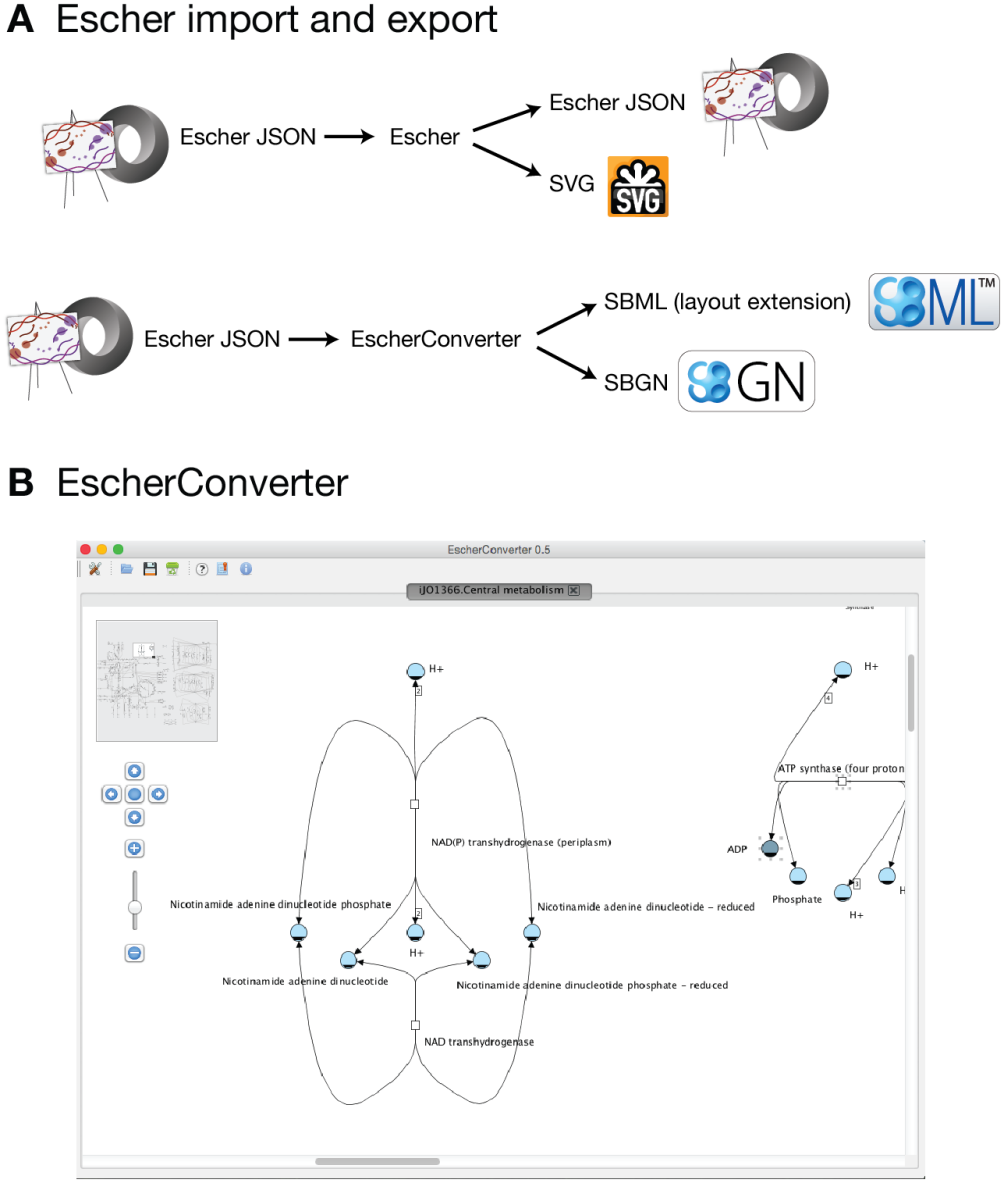
The import and export types in Escher and the EscherConverter. A) Escher can save to the Escher JSON file format or export to a SVG image. EscherConverter can be used to generate files in the SBML and SBGN-ML formats. B) The EscherConverter graphical user interface.

Escher represents biochemical reactions as transformations from a set of reactants to a set of products, and each reaction can be assigned enzymes using a Boolean *gene reaction rule*. Thus, Escher uses a well-defined representation of the biochemical network, but the scope of the Escher notation is much more specific than community standards such as Systems Biology Graphical Notation (SBGN) [34] and Systems Biology Markup Language (SBML) with the layout extension [35–37]. Escher can be exported to both formats using the EscherConverter application (Fig 4). EscherConverter is written in Java™, and it is available as a standalone executable file (S3 File) that includes a graphical user interface with graph drawing capabilities and a command-line interface. Files can be opened through drag and drop or the file menu, and a history of up to 10 recent files is stored. Several user preferences allow flexible customization of the file conversion. The conversion to SBML and SBGN-ML (the XML implementation of SBGN) relies heavily on JSBML [38] and libSBGN [39].

### Open-source development

Escher is hosted on GitHub, with a public bug tracker and tools for community contribution to the codebase (https://github.com/zakandrewking/escher). Documentation for Escher is available and was generated using Sphinx and ReadTheDocs (https://escher.readthedocs.org). This documentation includes a description of the Escher features and detailed information on the JavaScript and Python APIs.

### Integrating Escher with analysis workflows

The Escher Python package, which is available from the Python Package Index (PyPI, https://pypi.python.org), can be used to integrate Escher maps with data analysis and simulation workflows. Using the available functions, datasets can be applied to Escher maps, and the resulting maps can be saved as standalone web pages, saved as JSON or SVG, or exported using the EscherConverter as a command line utility. The Python package works directly with COBRA models using COBRApy [40]. It also includes functions for modifying all of the Escher map settings, including the color and size scales for all elements.

The Python package also includes a simple web server to run Escher locally. The web server caches maps and models for offline use, and users can also add maps to the cache directory so that they appear in the local web application. The following commands will install the package, print the location of the local cache directory, and run the Escher server:

# install escher
pip install escher
# print the cache directory
python -c “import escher; print escher.get_cache_dir()”
# run the local server (available at http://localhost:7778)
python -m escher.server

### Developing with Escher

Application programming interfaces (APIs) are available for both JavaScript and Python to enable users to build, modify, and export maps programmatically. The specific functions in the APIs are defined in the Escher Documentation. New web applications can be built on top of the basic Escher functions by developing with the Escher JavaScript API. The Documentation provides details on implementing a very simple web page with an embedded Escher map.

## Availability and Future Directions

Escher version 1.1 is now available. Bug fixes and new pathway maps will be released regularly, and a number of Escher applications are currently in progress. Escher releases will follow the Semantic Versioning guidelines (http://semver.org) so that application developers can rely on new versions of Escher to be backwards compatible.

### The Escher approach to web visualization

A major focus during development of future Escher versions will be to generalize and improve the approach to web visualization. As discussed in the Introduction, there are many types of biological visualizations that contribute to our interpretation of “omics” datasets. Successful user interface designs should be applicable to all of these visualization types, with modifications for the specific needs of a tool. As web platforms become ubiquitous for application development, it is important to consider what elements might be shared across a suite of visualization tools. This would make development of new tools easier, and improve interoperability between tools. For example, a genetic dataset in Escher could link directly to a visualization of the dataset on a genome browser.

### The BiGG database

Escher will be included in the next release of the BiGG database [33]. The BiGG database is a repository for COBRA models developed in the Systems Biology Research Group at the University of California, San Diego. BiGG already includes static pathway maps for many models in the database. Escher maps will be embedded in the web pages for models, reactions, and metabolites so that users can quickly see the network context of a biological component, and the maps will be available on both the BiGG and Escher websites.

### A community effort

The Escher framework is highly amenable to improvements, such as new visual features. Example improvements include compartment membranes, representations of regulation and signaling such as those in the SBGN specification, better statistical tools for analyzing and comparing various data types, more import and export options, and direct integration of other visualizations (such as protein and metabolite structures). Because Escher is an open-source project, contributions from the community—bug fixes, use cases, code contributions, etc.—will be encouraged and will be an important factor in making Escher a sustainable, long-term solution to the challenges of visualizing biological pathways.

## Availability and Requirements

1. Project name: Escher
2. Project home page: https://escher.github.io
3. Project source: https://github.com/zakandrewking/escher
4. Open-source license: MIT license
5. Operating systems(s): Platform independent
6. Programming languages: JavaScript, Python, and Java
7. Other requirements: none
8. Any restrictions to use by non-academics: no limitations

## Supporting Information

S1 File The source code for Escher JavaScript and Python libraries. This source code is for Escher version 1.1.2. The latest Escher source code can be cloned or downloaded from https://github.com/zakandrewking/escher.
(ZIP)

S2 File The Escher documentation as a PDF file. This documentation is for Escher version 1.1.2. The latest Escher documentation can be found at https://escher.readthedocs.org.
(ZIP)

S3 File The executable EscherConverter. Requires Java™ version 1.8 or higher. The latest version of EscherConverter is available on the Escher homepage at https://escher.github.io.
(ZIP)

S1 Data The *in silico* flux predictions used to demonstrate reaction data visualization. (ZIP)

S2 Data The metabolomics dataset used to demonstrate metabolite data visualization. (ZIP)

S3 Data The Escher map used to demonstrate metabolite data visualization. (ZIP)

S4 Data The transcriptomic dataset used to demonstrate gene data visualization. (ZIP)
