# Supplementary material for: Escher: A Web Application for Building, Sharing, and Embedding Data-Rich Visualizations of Biological Pathways
Source: PLoS Comput Biol. 2015 Aug 27;11(8):e1004321. doi: 10.1371/journal.pcbi.1004321 (PMC4552468; doi:10.1371/journal.pcbi.1004321)
Supplement: S1 File — This source code is for Escher version 1.1.2. The latest Escher source code can be cloned or downloaded from https://github.com/zakandrewking/escher. (ZIP) [file pcbi.1004321.s001.zip › escher-1.1.2/escher/templates/content.html]

{% if wrapper %}{% extends 'standalone.html' %}{% endif %}
{% block head %}


{% endblock %}
{% block content %}{% endblock %}
