# Supplementary material for: Escher: A Web Application for Building, Sharing, and Embedding Data-Rich Visualizations of Biological Pathways
Source: PLoS Comput Biol. 2015 Aug 27;11(8):e1004321. doi: 10.1371/journal.pcbi.1004321 (PMC4552468; doi:10.1371/journal.pcbi.1004321)
Supplement: S1 File — This source code is for Escher version 1.1.2. The latest Escher source code can be cloned or downloaded from https://github.com/zakandrewking/escher. (ZIP) [file pcbi.1004321.s001.zip › escher-1.1.2/escher/templates/index.html]

{% extends "standalone.html" %}
{% block title %}Escher{% endblock %}
{% block head %}


{% endblock %}
{% block content %}

# ESCHER

### Filter by organism

All

### Map

None

### Model (Optional)

None

### Tool

Builder
Viewer
{% if not web\_version and can\_dev %}
Builder (Dev)
Viewer (Dev)
{% endif %}

### Options

Scroll to zoom (instead of scroll to pan)

Never ask before reloading

Load map

---

## FAQ

1. #### What is Escher?

   Escher is a web-based tool for building, viewing, and sharing
   visualizations of biological pathways. These 'pathway maps' are a
   great way to contextualize data about metabolism. To get started,
   load a map by clicking the **Load Map** button above, or visit
   the documentation to learn more.
2. #### Are there more maps available? Can I contribute maps?

   We will be uploading maps for all of the organisms in the
   BiGG database. If you
   would like to contribute maps, there is a
   guide
   available in the documentation.
3. #### What browsers can I use?

   We recommend using Google Chrome for optimal performance, but
   Escher will also run in the latest versions of Firefox, Internet
   Explorer, and Safari (including mobile Safari).
4. #### I have more questions. Who do I ask?

   Visit the documentation
   to get started with Escher and explore the API. You can follow
   @zakandrewking
   for Escher updates. If you find bugs or would like to contribute
   to the project, feel free to submit an issue and or a pull request
   on Github.


Documentation – GitHub

Version {{version}}

{% endblock %}
