# Supplementary figures and images for: Escher: A Web Application for Building, Sharing, and Embedding Data-Rich Visualizations of Biological Pathways
Source: PLoS Comput Biol. 2015 Aug 27;11(8):e1004321. doi: 10.1371/journal.pcbi.1004321 (PMC4552468; doi:10.1371/journal.pcbi.1004321)

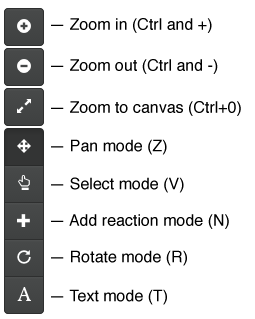

Supplement: S1 File — This source code is for Escher version 1.1.2. The latest Escher source code can be cloned or downloaded from https://github.com/zakandrewking/escher. (ZIP) [file pcbi.1004321.s001.zip › escher-1.1.2/docs/_static/bar.png]

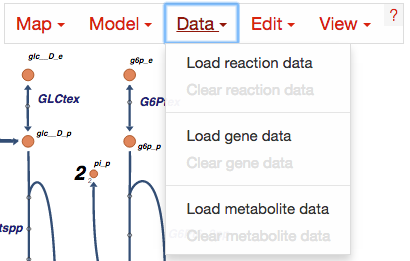

Supplement: S1 File — This source code is for Escher version 1.1.2. The latest Escher source code can be cloned or downloaded from https://github.com/zakandrewking/escher. (ZIP) [file pcbi.1004321.s001.zip › escher-1.1.2/docs/_static/data_menu.png]

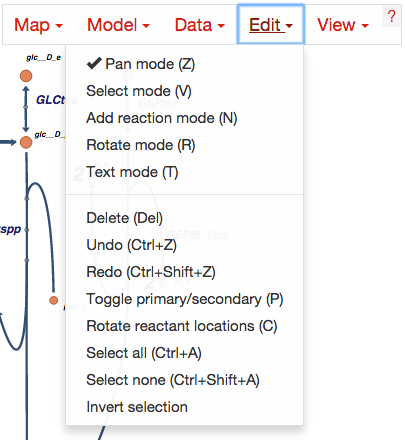

Supplement: S1 File — This source code is for Escher version 1.1.2. The latest Escher source code can be cloned or downloaded from https://github.com/zakandrewking/escher. (ZIP) [file pcbi.1004321.s001.zip › escher-1.1.2/docs/_static/edit_menu.png]

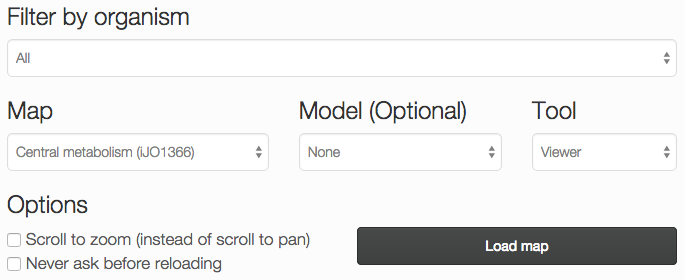

Supplement: S1 File — This source code is for Escher version 1.1.2. The latest Escher source code can be cloned or downloaded from https://github.com/zakandrewking/escher. (ZIP) [file pcbi.1004321.s001.zip › escher-1.1.2/docs/_static/launch_page.png]

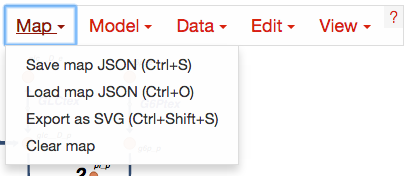

Supplement: S1 File — This source code is for Escher version 1.1.2. The latest Escher source code can be cloned or downloaded from https://github.com/zakandrewking/escher. (ZIP) [file pcbi.1004321.s001.zip › escher-1.1.2/docs/_static/map_menu.png]

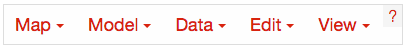

Supplement: S1 File — This source code is for Escher version 1.1.2. The latest Escher source code can be cloned or downloaded from https://github.com/zakandrewking/escher. (ZIP) [file pcbi.1004321.s001.zip › escher-1.1.2/docs/_static/menu.png]

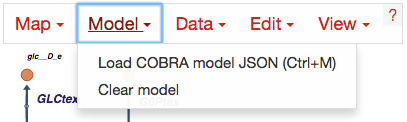

Supplement: S1 File — This source code is for Escher version 1.1.2. The latest Escher source code can be cloned or downloaded from https://github.com/zakandrewking/escher. (ZIP) [file pcbi.1004321.s001.zip › escher-1.1.2/docs/_static/model_menu.png]

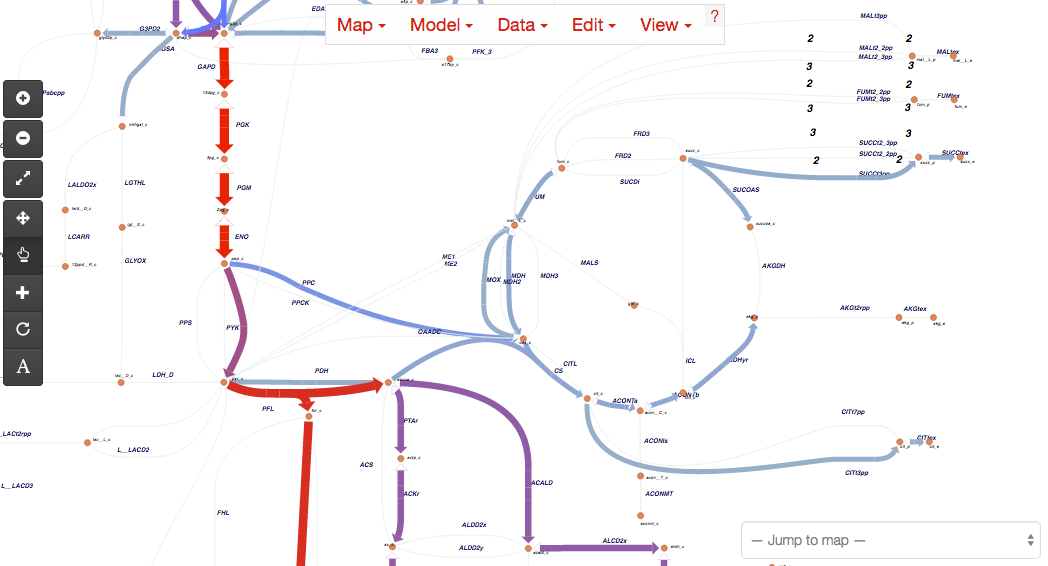

Supplement: S1 File — This source code is for Escher version 1.1.2. The latest Escher source code can be cloned or downloaded from https://github.com/zakandrewking/escher. (ZIP) [file pcbi.1004321.s001.zip › escher-1.1.2/docs/_static/screen1.png]

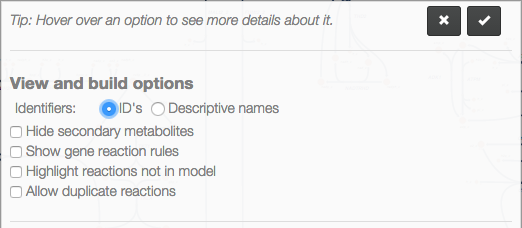

Supplement: S1 File — This source code is for Escher version 1.1.2. The latest Escher source code can be cloned or downloaded from https://github.com/zakandrewking/escher. (ZIP) [file pcbi.1004321.s001.zip › escher-1.1.2/docs/_static/settings.png]

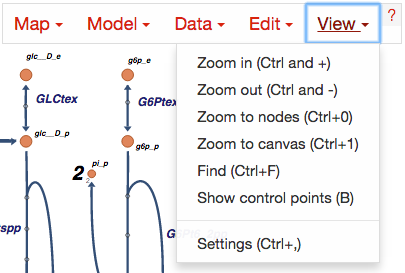

Supplement: S1 File — This source code is for Escher version 1.1.2. The latest Escher source code can be cloned or downloaded from https://github.com/zakandrewking/escher. (ZIP) [file pcbi.1004321.s001.zip › escher-1.1.2/docs/_static/view_menu.png]

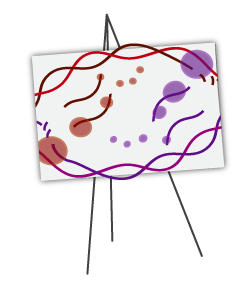

Supplement: S1 File — This source code is for Escher version 1.1.2. The latest Escher source code can be cloned or downloaded from https://github.com/zakandrewking/escher. (ZIP) [file pcbi.1004321.s001.zip › escher-1.1.2/escher/resources/escher-logo.png]

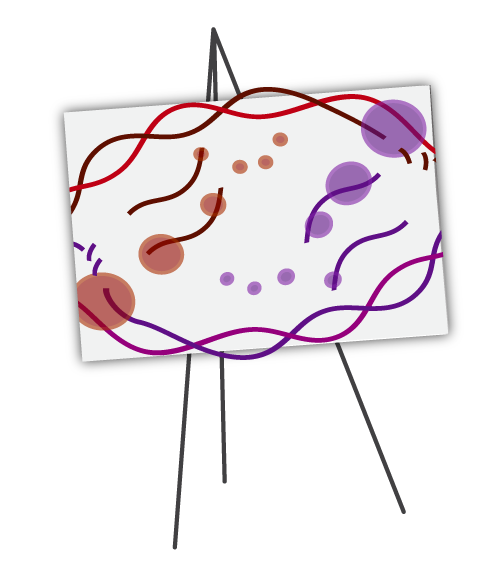

Supplement: S1 File — This source code is for Escher version 1.1.2. The latest Escher source code can be cloned or downloaded from https://github.com/zakandrewking/escher. (ZIP) [file pcbi.1004321.s001.zip › escher-1.1.2/escher/resources/escher-logo@2x.png]

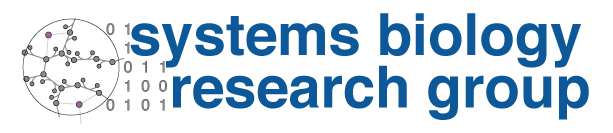

Supplement: S1 File — This source code is for Escher version 1.1.2. The latest Escher source code can be cloned or downloaded from https://github.com/zakandrewking/escher. (ZIP) [file pcbi.1004321.s001.zip › escher-1.1.2/escher/resources/sbrg-logo.png]
