# Supplementary material for: Escher: A Web Application for Building, Sharing, and Embedding Data-Rich Visualizations of Biological Pathways
Source: PLoS Comput Biol. 2015 Aug 27;11(8):e1004321. doi: 10.1371/journal.pcbi.1004321 (PMC4552468; doi:10.1371/journal.pcbi.1004321)
Supplement: S2 File — This documentation is for Escher version 1.1.2. The latest Escher documentation can be found at https://escher.readthedocs.org. (ZIP) [file pcbi.1004321.s002.zip › File S2 Escher Documentation.pdf]

Escher Documentation

Release 1.1.2

Zachary King

April 22, 2015

Contents

1 Help! I just upgraded to v1.0 and my maps will not load! **3**

2 Features **5**

3 Supported browsers **7**

4 Installation **9**

5 Contents **11**

5.1 Getting Started 11
5.2 Escher, COBRA, and COBRApy 19
5.3 Escher in the IPython Notebook 19
5.4 Convert Maps 20
5.5 Developing with Escher 21
5.6 Contributing Maps 21
5.7 JavaScript API 22
5.8 Python API 25

6 License **31**

Python Module Index **33**

Escher is a web-based tool for building, viewing, and sharing visualizations of biological pathways. These 'pathway maps' are a great way to contextualize biological datasets. The easiest way to use Escher is to browse and build maps on the Escher website. New users may be interested in the *Getting Started* guide.

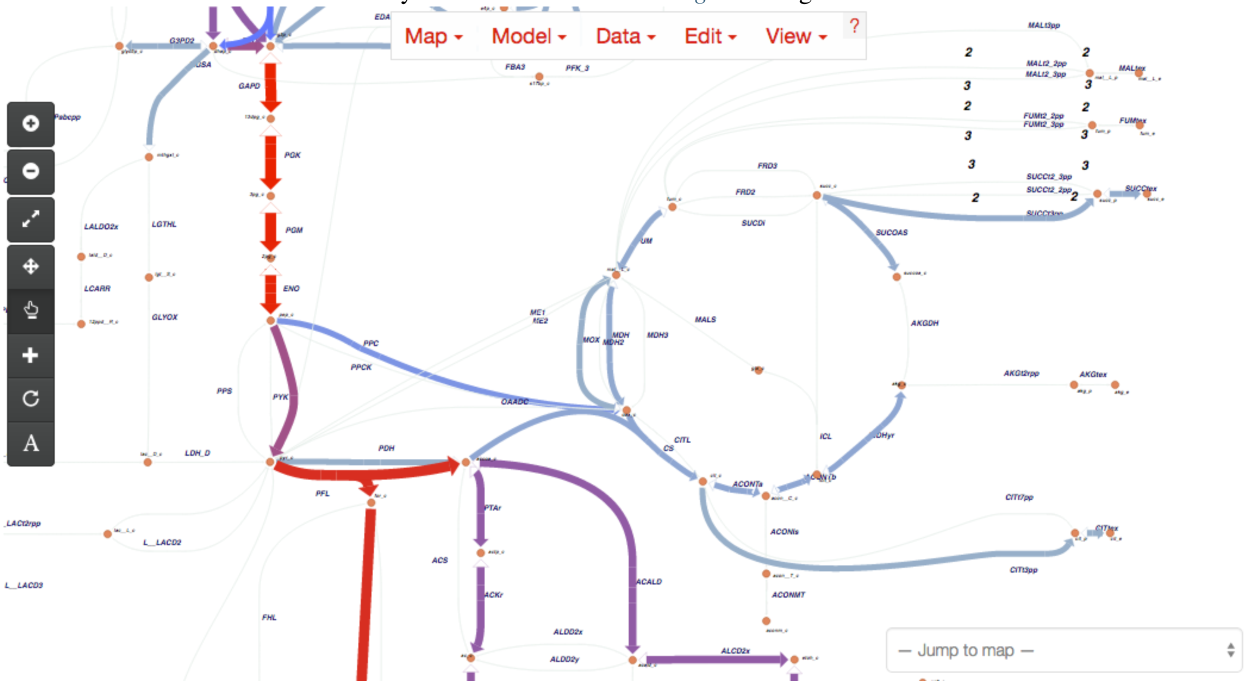

CHAPTER 1

Help! I just upgraded to v1.0 and my maps will not load!

If you have been using a pre-release version of Escher, you will need to convert your maps to the v1.0 format by following these *directions*. After version 1.0, the map format will not change until version 2.0.

CHAPTER 2

Features

1. View pathway maps in any modern web browser
2. *Build maps* using the content of genome-scale metabolic models
3. *Visualize data* on reactions, genes, and metabolites
4. Full text search
5. Detailed options for changing colors, sizes, and more, all from the web browser
6. View maps *inside the IPython Notebook*
7. *Embed maps* within any website, with minimal dependencies (escher.js, d3.js, and optionally Twitter Bootstrap)

CHAPTER 3

Supported browsers

We recommend using Google Chrome for optimal performance, but Escher will also run in the latest versions of Firefox, Internet Explorer, and Safari (including mobile Safari).

CHAPTER 4

Installation

Escher can be used without any installation by visiting the Escher website. However, you can install escher if you would like to (1) run Escher offline, (2) include your own maps and models in the launch page, (3) view Escher maps in an IPython Notebook, or (4) modify the source code.

To install the latest stable version of Escher, run:

```
pip install escher
```

For more information, see the documentation on *Escher in the IPython Notebook* and *Developing with Escher*.

CHAPTER 5

Contents

5.1 Getting Started

5.1.1 Introduction

Escher is here to help you visualize pathway maps. But, if you have never heard of a pathway map, you might appreciate a quick introduction.

What are pathway maps?

To understand pathway maps, it is useful to think about the general organization of a cell. At the smallest level, molecules in a cell are arranged in three-dimensional structures, and these structures determine many of the functions that take place in a cell. For example, the 3D structure of an enzyme determines the biochemical reactions that it can catalyze. These structures can be visualized in 3D using tools like Jmol (as in this example structure).

The DNA sequence is a second fundamental level of biological organization. DNA sequences are the blueprints for all the machinery of the cell, and they can be visualized as a one-dimensional series of bases (ATCG) using tools like the UCSC genome browser.

To use a football analogy, the 3D molecular structures are akin to the players on the field, and the information in the DNA sequence is like the playbook on the sidelines. But, football would not be very interesting if the players never took to the field and executed those plays. So, we are missing this level of detail: *the execution of biological plans by the molecular players*.

What we are missing is the biochemical reaction network. Proteins in the cell catalyze the conversion of substrate molecules into product molecules, and these *reactions* are responsible for generating energy, constructing cellular machinery and structures, detecting molecules in the environment, signaling, and more. Biochemical reactions can be grouped into pathways when they work in concert to carry out a function. (If a reaction is a football play, then the pathway is a drive). And Escher can be used to visualize these reactions and pathways. Together, we call these visualization **pathway maps**.

Escher to the rescue

Many Escher maps represent *metabolic* pathways, and Escher was developed at the Systems Biology Research Group where we have been building genome-scale models of metabolism over the past fifteen years. However, Escher is not limited to metabolism: It can be used to visualize any collection of biochemical reactions.

Escher includes one more killer feature: The ability to visualize datasets on a pathway map. Many biological discoveries are enabled by collecting and analyzing enormous datsets, and so biologists are grappling with the challenges of *big data*. By visualizing data in the context of pathway maps, we can quickly spot trends which would not be apparent

with standard statistical tools. And Escher visualizations can be adapted and shared to demonstrate those biological discoveries.

The rest of this guide will introduce the Escher user interface and the major features of Escher. You may also be interested in reading throught the `tutorials` for a more hands-on introduction to using Escher.

5.1.2 The launch page

When you open the Escher website, you will see a launch page that looks like this:

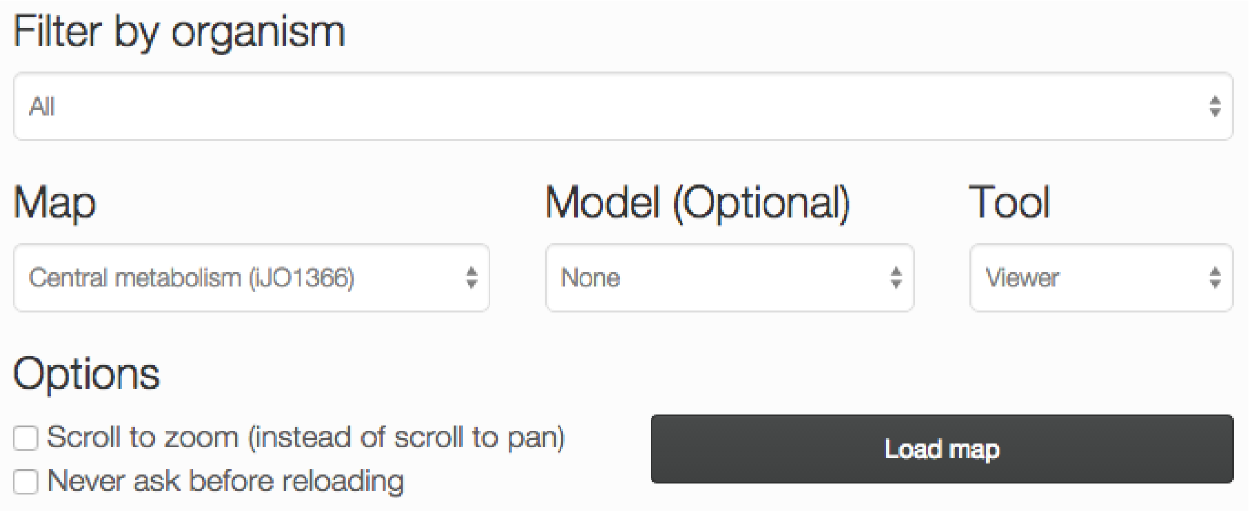

The options on the launch page are:

- *Filter by organism:* Choose an organism to filter the Maps and Models.
- *Map:* Choose a pre-built map, or start from scratch with an empty builder by choosing *None*. In parentheses next to the map name, you will see the name of the model that was used to build this map.
- *Model:* (Optional) If you choose a COBRA model to load, then you can add new reactions to the pathway map. You can also load your own model later, after you launch the tool. For an explanation of maps, models, and COBRA, see *Escher, COBRA, and COBRApy*.
- *Tool:*
  - The *Viewer* allows you to pan and zoom the map, and to visualize data for reactions, genes, and metabolites.
  - The *Builder*, in addition to the Viewer features, allows you to add reactions, move and rotate existing reactions, add text annotations, and adjust the map canvas.
- *Options:*
  - *Scroll to zoom (instead of scroll to pan)*: Determines the effect of using the mouse's scroll wheel over the map.
  - *Never ask before reloading*: If this is checked, then you will not be warned before leaving the page, even if you have unsaved changes.

Choose *Load map* to open the Escher viewer or builder in a new tab, and prepare to be delighted by your very own pathway map.

5.1.3 The menu bar

Once you have loaded an Escher map, you will see a menu bar along the top of the screen. Click the question mark to bring up the Escher documentation:

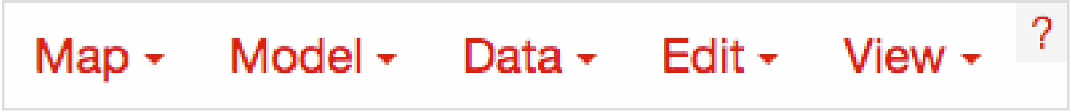

5.1.4 Loading and saving maps

Using the map menu, you can load and save maps at any time:

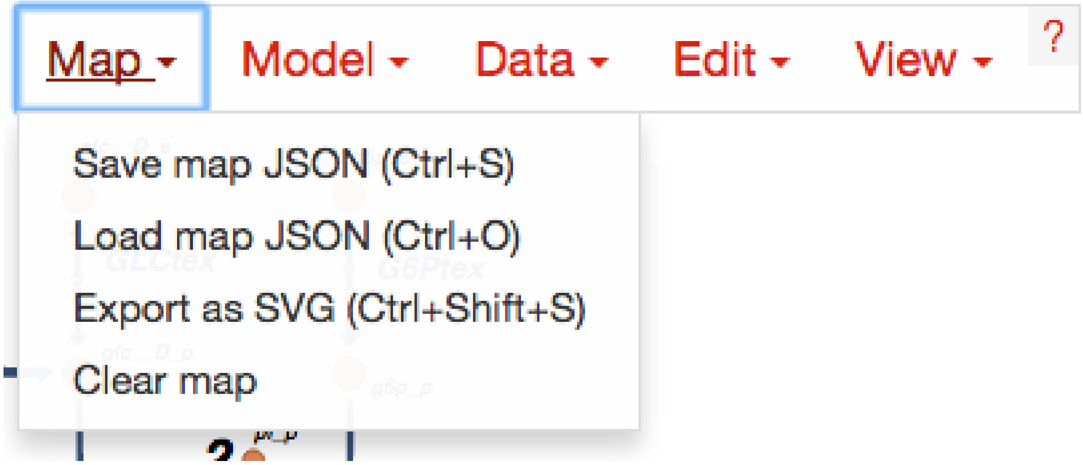

Click *Save map JSON* to save the Escher map as a JSON file, which is the standard file representing an Escher map. **NOTE**: The JSON file does NOT save any datasets you have loaded. This may be changed in a future version of Escher.

Later, you can can load a JSON file to view and edit a map by clicking *Load map JSON*.

Click *Export as SVG* to generate a SVG file for editing in tools like Adobe Illustrator and Inkscape. This is the best way to generate figures for presentations and publications. Unlike a JSON file, a SVG file maintains the data visualizations on the Escher map. However, you cannot load SVG files into Escher after you generate them.

Click *Clear Map* to empty the whole map, leaving a blank canvas. **NOTE**: You cannot undo *Clear Map*.

5.1.5 Loading models

Use the model menu to manage the COBRA model loaded in Escher:

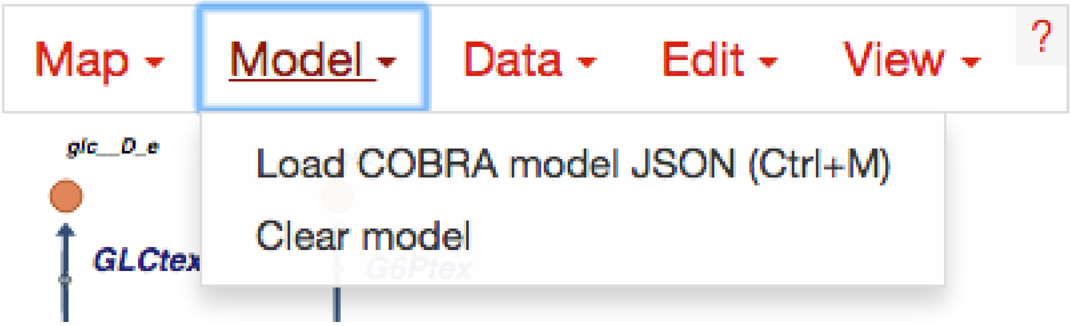

Choose *Load COBRA model JSON* to open a COBRA model. Read more about COBRA models in *Escher, COBRA, and COBRApy*. Once you have COBRApy v0.3.0 or later installed, then you can generate a JSON model by following this example code.

Click *Clear Model* to clear the current model.

5.1.6 Loading reaction, gene, and metabolite data

Datasets can be loaded as CSV files or JSON files, using the Data Menu.

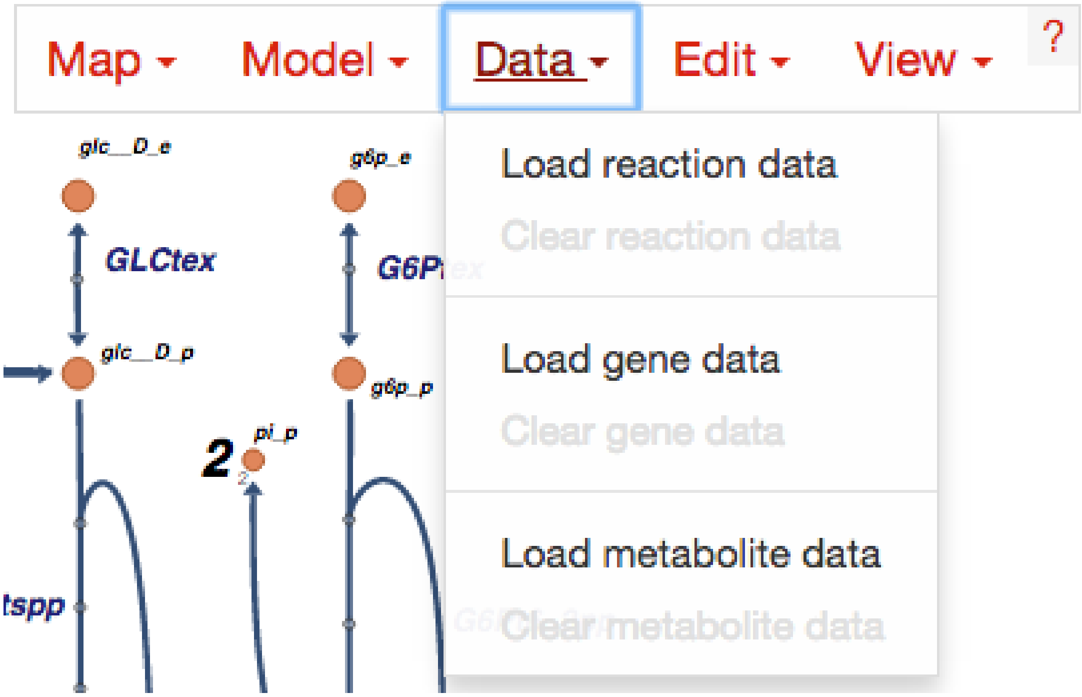

The structure of a CSV file

CSV files should have 1 header row, 1 ID column, and either 1 or 2 columns for data values. Here is an example with a single data value columns:

```
ID,time 0sec
glc__D_c,5.4
g6p__D_c,2.3
```

Which might look like this is Excel:

ID	time 0sec
glc__D_c	5.4
g6p_c	2.3

If two datasets are provided, then the Escher map will display the difference between the datasets. In the Settings menu, the **Comparison** setting allows you to choose between comparison functions (Fold Change, Log2(Fold Change), and Difference). With two datasets, the CSV file looks like this:

ID	time 0sec	time 5s
glc__D_c	5.4	10.2
g6p_c	2.3	8.1

Data can also be loaded from a JSON file. This Python code snippet provides an example of generating the proper format for single reaction data values and for reaction data comparisons:

```
import json

# save a single flux vector as JSON
flux_dictionary = {'glc__D_c': 5.4, 'g6p_c': 2.3}
with open('out.json', 'w') as f:
    json.dump(flux_dictionary, f)

# save a flux comparison as JSON
flux_comp = [{'glc__D_c': 5.4, 'g6p_c': 2.3}, {'glc__D_c': 10.2, 'g6p_c': 8.1}]
with open('out_comp.json', 'w') as f:
    json.dump(flux_comp, f)
```

Gene data and gene reaction rules

Escher uses *gene reaction rules* to connect gene data to the reactions on a metabolic pathway. You can see these gene reaction rules on the map by selecting *Show gene reaction rules* in the *Settings* menu.

Gene reaction rules show the genes whose gene products are required to catalyze a reaction. Gene are connected using AND and OR rules. AND rules are used when two genes are required for enzymatic activity, e.g. they are members of a protein complex. OR rules are used when either gene can catalyze the enzymatic activity, e.g. they are isozymes.

With OR rules, Escher will take the sum of the data values for each gene. With AND rules, Escher will either take the mean (the default) or the minimum of the components. The AND behavior (mean vs. minimum) is defined in the *Settings* menu.

You can look through the `tutorials` to see gene reaction rules in action.

5.1.7 Editing and building

The Edit menu gives you access to function for editing the map:

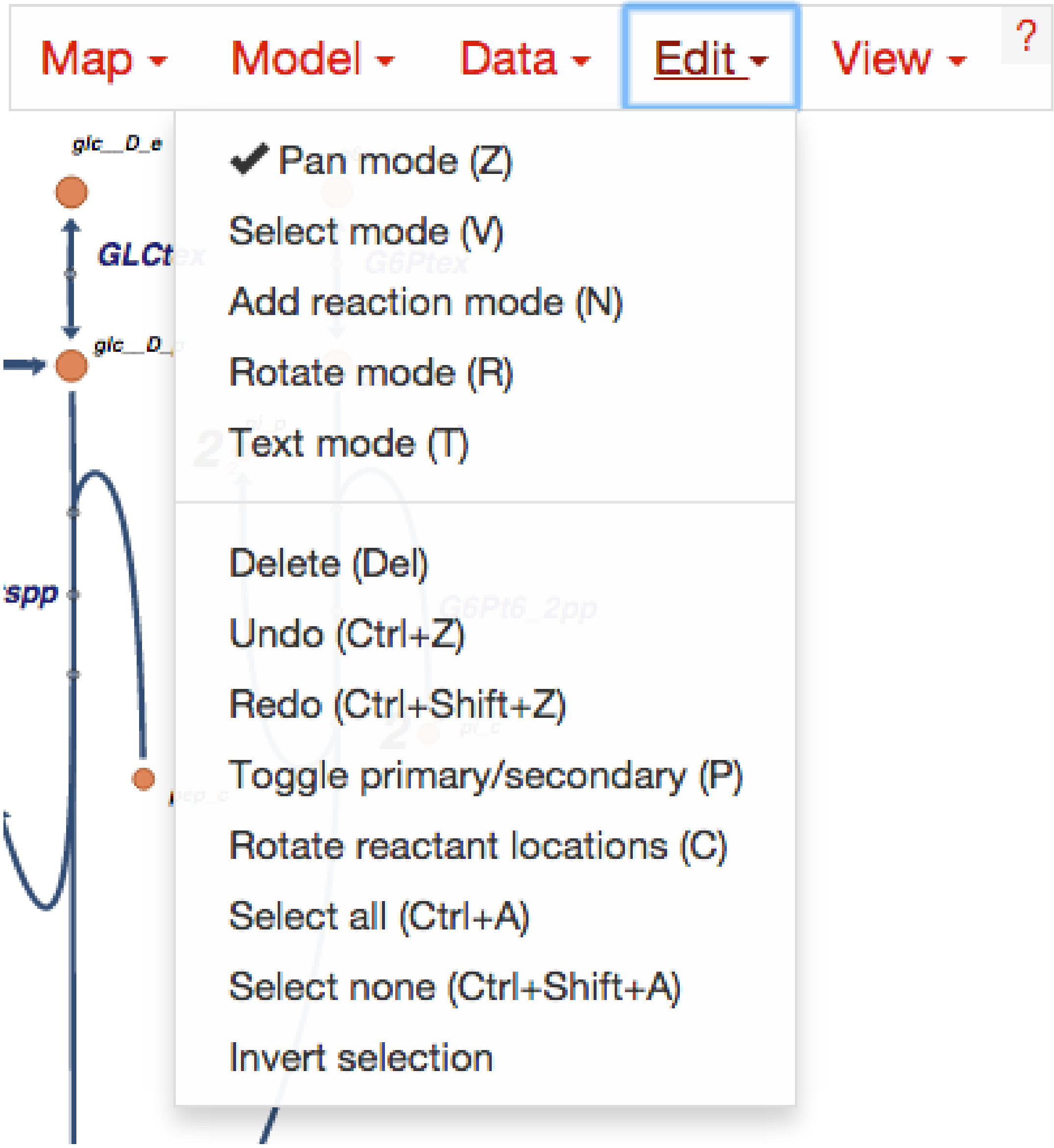

Escher has five major modes, and you can switch between those modes using the buttons in the Edit menu, or using the buttons in the *button bar* on the left of the screen.

5.1.8 View options

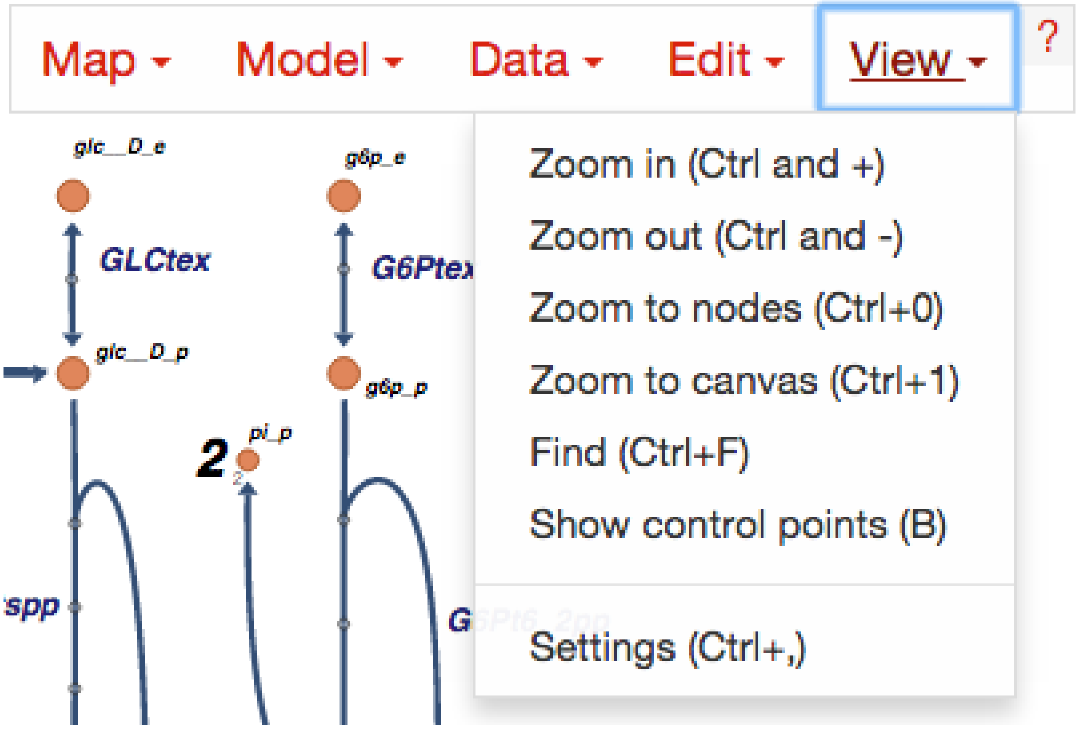

5.1.9 The button bar

The button bar give you quick access to many of the common Escher functions:

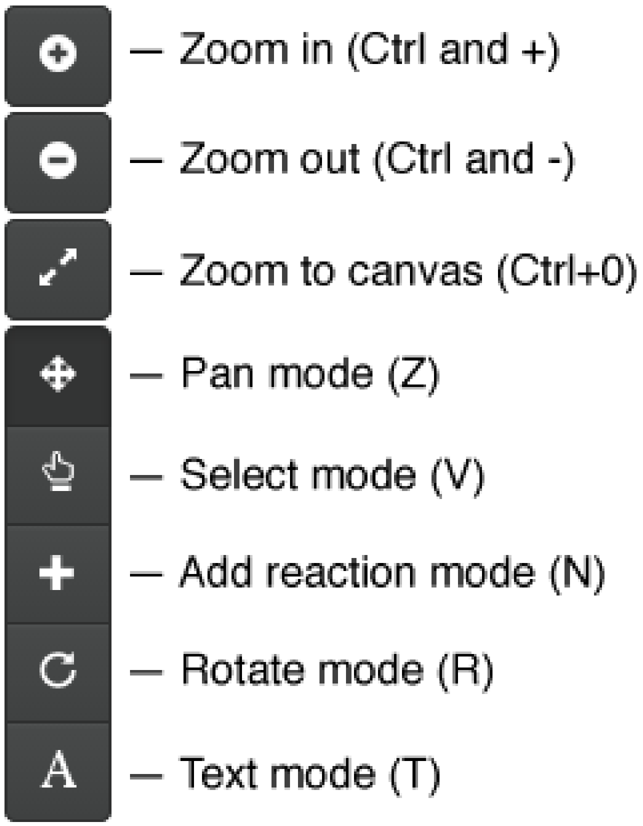

5.1.10 Settings

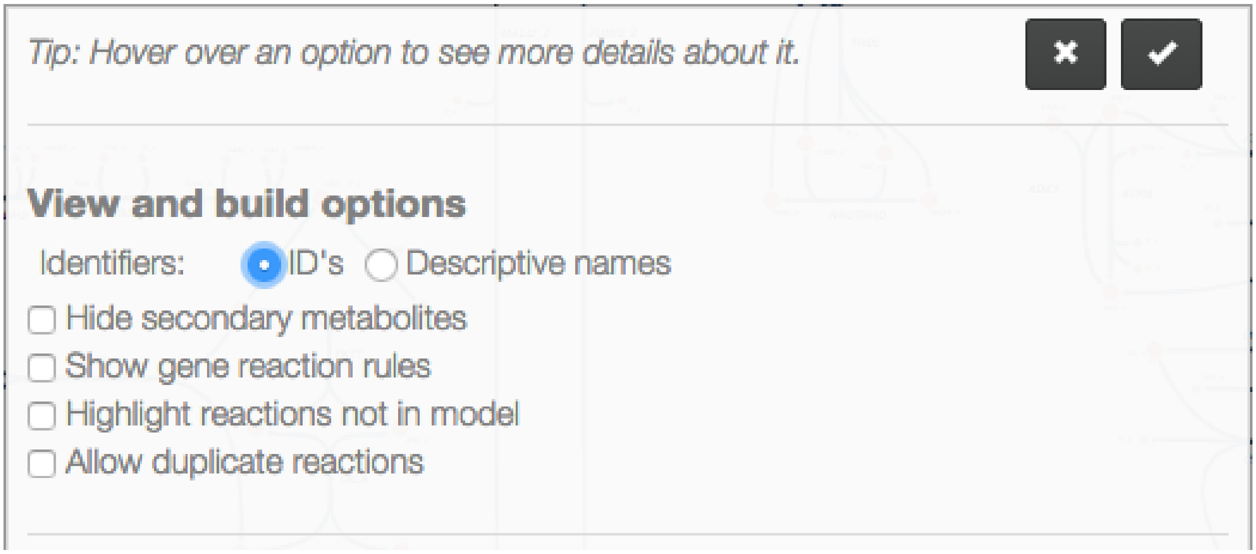

- *Identifiers:* Choose whether to show BiGG IDs or descriptive names for reactions, metabolites, and genes.
- *Show gene reaction rules:* Show the gene reaction rules below the reaction labels, even gene data is not loaded.
- *Highlight reactions not in model:* Highlight in red any reactions that are on the map but are not in the model. This is useful when you are adapting a map from one model for use with another model

- *Allow duplicate reactions:* By default, duplicate reactions are hidden in the add reaction dropdown menu. However, you can turn this option on to show the duplicate reactions.

5.2 Escher, COBRA, and COBRApy

Escher can be used as an independent application, but it draws heavily on the information in COBRA models. A COBRA model is a collection of all the reactions, metabolites, and genes known to exist in an organism (also called a genome-scale model (GEM) or constraint-based model (CBM)).

By loading a COBRA model into the Escher interface, you have access to every reaction and metabolite in that model. You also have the *gene reaction rules* for the reactions in the network, which allow you to connect gene data to reactions and metabolites.

COBRApy is a software package for COBRA modeling written in Python. The Escher Python package uses COBRApy package for reading and writing COBRA models.

5.2.1 Maps and models

In Escher, you will see references to *maps* and *models*.

A map contains the reactions and metabolites that you see in the Escher builder, including their locations, text annotations, and the canvas.

A model (a COBRA model) contains reactions and metabolites that you have not drawn yet. Thus, you can load a COBRA model when you want to draw new reactions on the map.

5.2.2 What is JSON and why do we use it?

Both Escher maps and COBRA models are stored as JSON files. JSON is a useful, plain-text format for storing nested data structures. We use JSON much like the SBML community uses XML. You may notice that SBML files have a .xml extension, and Escher maps and COBRA models have a .json extension.

You can use Python to explore a JSON file like this:

```python
import json

with open('map.json', 'r') as f:
    map_object = json.load(f)

print map_object[0]
print map_object[1]['reactions'].values()[0]
```

5.2.3 Escher, SBML, and SBGN

A tool has been developed for converting Escher maps to SBML Layout and SBGN, and it will be released soon.

COBRA models can be converted to SBML using COBRApy.

5.3 Escher in the IPython Notebook

The Python package for Escher can be installed using pip:

```
pip install escher
```

Alternatively, one can download the source files and install the package directly:

```
python setup.py install
```

Once you have installed Escher locally, you can interact with Escher maps in an IPython Notebook.

Here are example notebooks to get started with:

- COBRApy and Escher
- JavaScript development and offline maps
- Generate JSON models in COBRApy

Dependencies:

- Jinja2
- Tornado
- COBRApy, 0.3.0 or later

5.4 Convert Maps

Any Escher maps built with pre-release versions of Escher will not load right away in the stable v1.0 release. To convert pre-release maps to the new format, follow these steps:

1. Install Escher:

   ```
   pip install escher
   ```

2. Find a COBRA model for your maps. This COBRA model will be used to update the content of the map in order to support all the new Escher features. You can use a COBRA model encoded as SBML or JSON (generated with COBRApy v0.3.0b4 or later). The COBRA models currently available on the Escher website can be downloaded here:

https://github.com/escher/escher.github.io/tree/master/1-0-0/models

   For a refresher on the distinction between Escher maps, COBRA models, and their file types (SBML, JSON, SBML Layout), see *Escher, COBRA, and COBRApy*.

3. Run the `convert_map` script to convert your existing Escher map (my_old_map.json) to the new format, using a COBRA model (model_file.json or model_file.xml in these examples):

   ```
   # With a JSON file model
   python -m escher.convert_map my_old_map.json path/to/model_file.json

   # With an SBML model
   python -m escher.convert_map my_old_map.json path/to/model_file.xml
   ```

Those commands will generate a new map called `my_old_map_converted.json` that will load in Escher v1.0 and later.

If that doesn't work, and you were a Beta user for Escher v1, you can DM @zakandrewking with a link to a broken Escher map (using Pastbin, Dropbox, or similar) and the COBRA model you used to generate the map. As a thank you for helping out with Escher development, I will run the `convert_map` script and send you back a new, valid map. -Zak

5.5 Developing with Escher

5.5.1 Using the static javascript files

You can include the compiled Escher javascript file in any html document. The only dependencies are d3.js, and Twitter Bootstrap if you are using the option menu='all'. These files can be found in `escher/lib`.

For an example of the boilerplate code that is required to begin developing with Escher, have a look at the escher-demo repository.

5.5.2 Running the local server

You can run your own local server if you want to modify the Escher code, or use Escher offline. To get started, install the Python package and run from any directory by calling:

```
python -m escher.server
```

This starts a server at localhost:7778. You can also choose another port:

```
python -m escher.server --port=8005
```

5.5.3 Building and testing Escher

Build the minified and non-minified javascript files:

```
python setup.py buildjs
```

Test Python and start Jasmine for JavaScript testing:

```
python setup.py test
```

Build the static website:

```
python setup.py buildgh
```

Clear static website files:

```
python setup.py clean
```

5.5.4 The Escher file format

5.6 Contributing Maps

If you would like to contribute maps to Escher, you can make a Pull Request to the GitHub repository escher.github.io. Make sure there is a folder with the name of the organism in `1-0-0/maps`. For example, a new yeast map goes in the folder:

```
1-0-0/maps/Saccharomyces cerevisiae/
```

Then, name your map by concatenating the model ID and the map name, separated by a period. For example, a yeast map built with the genome-scale model iMM904 could be named:

```
iMM904.Amino acid biosynthesis.json
```

Then, add the JSON file for the model to the Pull Request *if that model is not already available*. As before, make a folder for your organism within `1-0-0/models/`. The model filename is just the model ID.

In this example, a correct Pull Request would include the following files:

```
1-0-0/maps/Saccharomyces cerevisiae/iMM904.Amino acid biosynthesis.json
1-0-0/models/Saccharomyces cerevisiae/iMM904.json
```

5.7 JavaScript API

class `escher.`**`Builder`**(*map_data*, *model_data*, *embedded_css*, *options*)

A Builder object contains all the UI and logic to generate a map builder or viewer.

Arguments

- **map_data** (*object*) – The data for a map, to be passed to escher.Map.from_data(). If null, then an empty Builder is initialized
- **model_data** (*object*) – The data for a cobra model, to be passed to escher.CobraModel(). Can be null.
- **embedded_css** (*string*) – The stylesheet for the SVG elements in the Escher map.
- **selection** (*object*) – (Optional, Default: In the body element) The d3 selection of an element to place the Builder into. The selection cannot be inside an SVG element.
- **options** (*object*) – (Optional) An object defining any of the following options:

`options.`**`menu`**

(Default: 'all') The type of menu that will be displayed. Can be 'all' for the full menu or 'zoom' for just zoom buttons. The 'all' option requires the full set of Escher dependencies (D3.js, JQuery, and Bootstrap), while the 'zoom' option requires only D3.js. For more details, see *Developing with Escher*.

`options.`**`scroll_behavior`**

(Default: 'pan') This option determines the effect that the scroll wheel will have on an Escher map. Can be 'pan' to pan the map or 'zoom' to zoom the map when the user moves the scroll wheel.

`options.`**`enable_editing`**

(Default: true) If true then display the map editing functions. If false, then hide them and only allow the user to view the map.

`option.`**`enable_keys`**

(Default: true) If true then enable keyboard shortcuts.

`options.`**`enable_search`**

(Default: true) If true, then enable indexing of the map for search. Use false to disable searching and potentially improve the map performance.

`options.`**`fill_screen`**

(Default: false) Use true to fill the screen when an Escher Builder is placed in a top-level container (e.g. a div in the body element).

Map, model, and styles

`options.`**`starting_reaction`**

(Default: null) The ID (as a string) of a reaction to draw when the Builder loads.

options.**never_ask_before_quit**
(Default: false) If false, then display a warning before the user closes an Escher map. If true, then never display the warning. This options is only respected if options.enable_editing == true. If enable_editing is false, then the warnings are not displayed.

options.**unique_map_id**
(Default: null) A unique ID that will be used to UI elements don't interfere when multiple maps are in the same HTML document.

options.**primary_metabolite_radius**
(Default: 15) The radius of primary metabolites, in px.

options.**secondary_metabolite_radius**
(Default: 10) The radius of secondary metabolites, in px.

options.**marker_radius**
(Default: 5) The radius of marker nodes, in px.

options.**gene_font_size**
(Default: 18) The font size of the gene reaction rules, in px.

options.**hide_secondary_metabolites**
(Default: false) If true, then secondary nodes and segments are hidden. This is convenient for generating simplified map figures.

options.**show_gene_reaction_rules**
(Default: false) If true, then show the gene reaction rules, even without gene data.

Applied data

options.**reaction_data**
(Default: null) An object with reaction ids for keys and reaction data points for values.

options.**reaction_styles**
(Default: ['color', 'size', 'text']) An array of style types. The array can contain any of the following: 'color', 'size', 'text', 'abs'. The 'color' style means that the reactions will be colored according to the loaded dataset. The 'size' style means that the reactions will be sized according to the loaded dataset. The 'text' style means that the data values will be displayed in the reaction labels. The 'abs' style means the the absolute values of reaction values will be used for data visualization.

options.**reaction_compare_style**
(Default: 'diff') How to compare to datasets. Can be either 'fold, 'log2_fold', or 'diff'.

options.**reaction_scale**
(Default: [{ type: 'min', color: '#c8c8c8', size: 12 }, { type: 'median', color: '#9696ff', size: 20 }, { type: 'max', color: '#ff0000', size: 25 }])

options.**reaction_no_data_color**
(Default: '#dcdcdc') The color of reactions with no data value.

options.**reaction_no_data_size**
(Default: 8) The size of reactions with no data value.

options.**gene_data**
(Default: null) An object with Gene ids for keys and gene data points for values.

options.**and_method_in_gene_reaction_rule**
(Default: mean) When evaluating a gene reaction rule, use this function to evaluate AND rules. Can be 'mean' or 'min'.

options.**metabolite_data**
(Default: null) An object with metabolite ids for keys and metabolite data points for values.

options.**metabolite_styles**
(Default: ['color', 'size', 'text']) An array of style types. The array can contain any of the following: 'color', 'size', 'text', 'abs'. The 'color' style means that the metabolites will be colored according to the loaded dataset. The 'size' style means that the metabolites will be sized according to the loaded dataset. The 'text' style means that the data values will be displayed in the metabolite labels. The 'abs' style means the the absolute values of metabolite values will be used for data visualization.

options.**metabolite_compare_style**
(Default: 'diff') How to compare to datasets. Can be either 'fold', 'log2_fold' or 'diff'.

options.**metabolite_scale**
(Default: [{ type: 'min', color: '#fffaf0', size: 20 }, { type: 'median', color: '#f1c470', size: 30 }, { type: 'max', color: '#800000', size: 40 }])

options.**metabolite_no_data_color**
(Default: '#ffffff') The color of metabolites with no data value.

options.**metabolite_no_data_size**
(Default: 10) The size of metabolites with no data value.

View and build options

options.**identifiers_on_map**
(Default: 'bigg_id') The identifiers that will be displayed in reaction, metabolite, and gene labels. Can be 'bigg_id' or 'name'.

options.**highlight_missing**
(Default: false) If true, then highlight in red reactions that are not in the loaded COBRA model.

options.**allow_building_duplicate_reactions**
(Default: true) If true, then building duplicate reactions is allowed. If false, then duplicate reactions are hidden in *Add reaction mode*.

Callbacks

options.**first_load_callback**
A function to run after loading the Builder.

load_map(*map_data*[, *should_update_data*])
Load a map for the loaded data. Also reloads most of the Builder content.

Arguments

- **map_data** – The data for a map.
- **should_update_data** (*Boolean*) – (Default: true) Whether data should be applied to the map.

load_model(*model_data*[, *should_update_data*])
Load the cobra model from model data.

Arguments

- **model_data** – The data for a Cobra model. (Parsing in done by escher.CobraModel).
- **should_update_data** (*Boolean*) – (Default: true) Whether data should be applied to the model.

view_mode()
Enter view mode.

build_mode()
Enter build mode.

brush_mode()
Enter brush mode.

zoom_mode()
Enter zoom mode.

rotate_mode()
Enter rotate mode.

text_mode()
Enter text mode.

set_reaction_data(*data*)

Arguments

- **data** (*array*) – An array of 1 or 2 objects, where each object has keys that are reaction ID's and values that are data points (numbers).

set_metabolite_data(*data*)

Arguments

- **data** (*array*) – An array of 1 or 2 objects, where each object has keys that are metabolite ID's and values that are data points (numbers).

set_gene_data(*data*, *clear_gene_reaction_rules*)

Arguments

- **data** (*array*) – An array of 1 or 2 objects, where each object has keys that are gene ID's and values that are data points (numbers).
- **clear_gene_reaction_rules** (*Boolean*) – (Optional, Default: false) In addition to setting the data, also turn of the gene_reaction_rules.

5.8 Python API

class escher.**Builder**(*map_name=None*, *map_json=None*, *model=None*, *model_name=None*, *model_json=None*, *embedded_css=None*, *reaction_data=None*, *metabolite_data=None*, *gene_data=None*, *local_host=None*, *id=None*, *safe=False*, ***kwargs*)

A metabolic map that can be viewed, edited, and used to visualize data.

This map will also show metabolic fluxes passed in during consruction. It can be viewed as a standalone html inside a browswer. Alternately, the respresentation inside an IPython notebook will also display the map.

Maps are stored in json files and are stored in a cache directory. Maps which are not found will be downloaded from a map repository if found.

Parameters

- **map_name** – A string specifying a map to be downloaded from the Escher web server, or loaded from the cache.

- **map_json** – A JSON string, or a file path to a JSON file, or a URL specifying a JSON file to be downloaded.
- **model** – A Cobra model.
- **model_name** – A string specifying a model to be downloaded from the Escher web server, or loaded from the cache.
- **model_json** – A JSON string, or a file path to a JSON file, or a URL specifying a JSON file to be downloaded.
- **embedded_css** – The CSS (as a string) to be embedded with the Escher SVG.
- **reaction_data** – A dictionary with keys that correspond to reaction ids and values that will be mapped to reaction arrows and labels.
- **metabolite_data** – A dictionary with keys that correspond to metabolite ids and values that will be mapped to metabolite nodes and labels.
- **gene_data** – A dictionary with keys that correspond to gene ids and values that will be mapped to corresponding reactions.
- **local_host** – A hostname that will be used for any local files in dev mode.
- **id** – Specify an id to make the javascript data definitions unique. A random id is chosen by default.
- **safe** – If True, then loading files from the filesytem is not allowed. This is to ensure the safety of using Builder with a web server.

Keyword Arguments

These are defined in the Javascript API:

- identifiers_on_map
- show_gene_reaction_rules
- unique_map_id
- primary_metabolite_radius
- secondary_metabolite_radius
- marker_radius
- hide_secondary_metabolites
- reaction_styles
- reaction_compare_style
- reaction_scale
- reaction_no_data_color
- reaction_no_data_size
- and_method_in_gene_reaction_rule
- metabolite_styles
- metabolite_compare_style
- metabolite_scale
- metabolite_no_data_color
- metabolite_no_data_size

- highlight_missing
- allow_building_duplicate_reactions

All keyword arguments can also be set on an existing Builder object using setter functions, e.g.:

```
my_builder.set_reaction_styles(new_styles)
```

display_in_browser(*ip=u'127.0.0.1'*, *port=7655*, *n_retries=50*, *js_source=u'web'*, *menu=u'all'*, *scroll_behavior=u'pan'*, *enable_editing=True*, *enable_keys=True*, *minified_js=True*, *never_ask_before_quit=False*)

Launch a web browser to view the map.

Parameters

- **ip** – The IP address to serve the map on.
- **port** – The port to serve the map on. If specified the port is occupied, then a random free port will be used.
- **n_retries** (*int*) – The number of times the server will try to find a port before quitting.
- **js_source** (*string*) – Can be one of the following:
  - *web* (Default) - Use JavaScript files from escher.github.io.
  - *local* - Use compiled JavaScript files in the local Escher installation. Works offline.
  - *dev* - Use the local, uncompiled development files. Works offline.
- **menu** (*string*) – Menu bar options include:
  - *none* - No menu or buttons.
  - *zoom* - Just zoom buttons.
  - *all* (Default) - Menu and button bar (requires Bootstrap).
- **scroll_behavior** (*string*) – Scroll behavior options:
  - *pan* - Pan the map.
  - *zoom* - Zoom the map.
  - *none* (Default) - No scroll events.
- **enable_editing** (*Boolean*) – Enable the map editing modes.
- **enable_keys** (*Boolean*) – Enable keyboard shortcuts.
- **minified_js** (*Boolean*) – If True, use the minified version of js files. If js_source is *dev*, then this option is ignored.
- **never_ask_before_quit** (*Boolean*) – Never display an alert asking if you want to leave the page. By default, this message is displayed if enable_editing is True.

display_in_notebook(*js_source=u'web'*, *menu=u'zoom'*, *scroll_behavior=u'none'*, *minified_js=True*, *height=500*)

Embed the Map within the current IPython Notebook.

Parameters

- **js_source** (*string*) – Can be one of the following:
  - *web* (Default) - Use JavaScript files from escher.github.io.
  - *local* - Use compiled JavaScript files in the local Escher installation. Works offline.
  - *dev* - Use the local, uncompiled development files. Works offline.

- **menu** (*string*) – Menu bar options include:
  - *none* - No menu or buttons.
  - *zoom* - Just zoom buttons.
  - Note: The *all* menu option does not work in an IPython notebook.
- **scroll_behavior** (*string*) – Scroll behavior options:
  - *pan* - Pan the map.
  - *zoom* - Zoom the map.
  - *none* - (Default) No scroll events.
- **minified_js** (*Boolean*) – If True, use the minified version of js files. If js_source is *dev*, then this option is ignored.
- **height** – Height of the HTML container.

save_html (*filepath=None*, *js_source=u'web'*, *menu=u'all'*, *scroll_behavior=u'pan'*, *enable_editing=True*, *enable_keys=True*, *minified_js=True*, *never_ask_before_quit=False*, *static_site_index_json=None*)

Save an HTML file containing the map.

Parameters

- **filepath** (*string*) – The HTML file will be saved to this location.
- **js_source** (*string*) – Can be one of the following:
  - *web* (Default) - Use JavaScript files from escher.github.io.
  - *local* - Use compiled JavaScript files in the local Escher installation. Works offline.
  - *dev* - Use the local, uncompiled development files. Works offline.
- **menu** (*string*) – Menu bar options include:
  - *none* - No menu or buttons.
  - *zoom* - Just zoom buttons.
  - *all* (Default) - Menu and button bar (requires Bootstrap).
- **scroll_behavior** (*string*) – Scroll behavior options:
  - *pan* - Pan the map.
  - *zoom* - Zoom the map.
  - *none* (Default) - No scroll events.
- **enable_editing** (*Boolean*) – Enable the map editing modes.
- **enable_keys** (*Boolean*) – Enable keyboard shortcuts.
- **minified_js** (*Boolean*) – If True, use the minified version of js files. If js_source is *dev*, then this option is ignored.
- **height** (*number*) – Height of the HTML container.
- **never_ask_before_quit** (*Boolean*) – Never display an alert asking if you want to leave the page. By default, this message is displayed if enable_editing is True.
- **static_site_index_json** (*string*) – The index, as a JSON string, for the static site. Use javascript to parse the URL options. Used for generating static pages (see static_site.py).

5.8.1 Cache

`escher.get_cache_dir(name=None)`
Get the cache dir as a string.

Parameters **name** (*string*) – An optional subdirectory within the cache

`escher.clear_cache(different_cache_dir=None)`
Empty the contents of the cache directory.

Parameters **different_cache_dir** (*string*) – (Optional) The directory of another cache. This is mainly for testing.

`escher.list_cached_maps()`
Return a list of all cached maps.

`escher.list_cached_models()`
Return a list of all cached models.

`escher.list_available_maps()`
Return a list of all maps available on the server

`escher.list_available_models()`
Return a list of all models available on the server

- *genindex*

CHAPTER 6

License

Escher is licensed under the MIT license.

Python Module Index

e

escher, 25

B

brush_mode() (built-in function), 25
build_mode() (built-in function), 25
Builder (class in escher), 25

C

clear_cache() (in module escher), 29

D

display_in_browser() (escher.Builder method), 27
display_in_notebook() (escher.Builder method), 27

E

escher (module), 25
escher.Builder() (class), 22

G

get_cache_dir() (in module escher), 29

L

list_available_maps() (in module escher), 29
list_available_models() (in module escher), 29
list_cached_maps() (in module escher), 29
list_cached_models() (in module escher), 29
load_map() (built-in function), 24
load_model() (built-in function), 24

O

option.enable_keys (option attribute), 22
options.allow_building_duplicate_reactions (options attribute), 24
options.and_method_in_gene_reaction_rule (options attribute), 23
options.enable_editing (options attribute), 22
options.enable_search (options attribute), 22
options.fill_screen (options attribute), 22
options.first_load_callback (options attribute), 24
options.gene_data (options attribute), 23
options.gene_font_size (options attribute), 23
options.hide_secondary_metabolites (options attribute), 23
options.highlight_missing (options attribute), 24
options.identifiers_on_map (options attribute), 24
options.marker_radius (options attribute), 23
options.menu (options attribute), 22
options.metabolite_compare_style (options attribute), 24
options.metabolite_data (options attribute), 23
options.metabolite_no_data_color (options attribute), 24
options.metabolite_no_data_size (options attribute), 24
options.metabolite_scale (options attribute), 24
options.metabolite_styles (options attribute), 24
options.never_ask_before_quit (options attribute), 22
options.primary_metabolite_radius (options attribute), 23
options.reaction_compare_style (options attribute), 23
options.reaction_data (options attribute), 23
options.reaction_no_data_color (options attribute), 23
options.reaction_no_data_size (options attribute), 23
options.reaction_scale (options attribute), 23
options.reaction_styles (options attribute), 23
options.scroll_behavior (options attribute), 22
options.secondary_metabolite_radius (options attribute), 23
options.show_gene_reaction_rules (options attribute), 23
options.starting_reaction (options attribute), 22
options.unique_map_id (options attribute), 23

R

rotate_mode() (built-in function), 25

S

save_html() (escher.Builder method), 28
set_gene_data() (built-in function), 25
set_metabolite_data() (built-in function), 25
set_reaction_data() (built-in function), 25

T

text_mode() (built-in function), 25

V

view_mode() (built-in function), 24

Z

zoom_mode() (built-in function), 25
